# Assessment of Diabetic Patients' Adherence to Diabetic Retinopathy Screening and the Influencing Factors in Al-Ahsa, Saudi Arabia

**DOI:** 10.7759/cureus.28253

**Published:** 2022-08-22

**Authors:** Abdulaziz Al Taisan, Adi Mohammed Al Owaifeer, Noor Al Osaif, Ali A Al Saeed, Bashayer F Al Furaikh, Lamis F AlJamaan

**Affiliations:** 1 Department of Surgery, College of Medicine, King Faisal University, Al-Ahsa, SAU; 2 Glaucoma Division, King Khaled Eye Specialist Hospital, Riyadh, SAU; 3 Department of Ophthalmology, College of Medicine, King Faisal University, Al-Ahsa, SAU; 4 Department of Pediatric Surgery, Maternity and Children Hospital, Dammam, SAU; 5 Ministry of Health, Al-Ahsa, SAU; 6 Department of Medicine, King Faisal University, Al Hofuf, SAU; 7 Department of Pediatrics, King Abdulaziz Medical City - National Guard Health Affairs, Al-Ahsa, SAU

**Keywords:** saudi arabia, al ahsaa, barriers, diabetic retinopathy screening, diabetic retinopathy

## Abstract

Background

Diabetic retinopathy (DR) is one of the diabetic complications that leads to the loss of vision. Most diabetic patients will have DR that is varying in its severity under the effect of many factors such as type of diabetes, duration of diabetes, and poor glycemic control. This study aimed to assess the level of adherence of diabetic patients to diabetic retinopathy screening (DRS) and to identify the influencing factors of adherence among diabetic patients in Al Ahsa, Saudi Arabia.

Methods

A cross-sectional study was conducted via a self-administered questionnaire among diabetic patients who were attending the primary health-care centers in Al Ahsa, Saudi Arabia, from July to August 2021. This questionnaire included five sections: sociodemographic data, diabetic profile, assessment of the knowledge, attitude toward DRS, and barriers to DRS.

Results

A total of 397 diabetic patients were involved in the study. Diabetic ocular complications were reported among 35.3% of the patients. The most commonly reported diabetic eye complication was cataract (37.1%; 52) followed by retinopathy (36.4%). Among the participants, 32.2% had a good awareness level regarding DR. In addition, 46.9% of diabetic patients have DRS. The most reported barriers were having no visual or eye problems, difficulty getting an appointment, and the cost and lack of information about the screening procedure (50.9%, 50.1%, 42.1%, and 39.8%, respectively).

Conclusion

Improvement of patients’ knowledge is a significant step to enhancing adherence to DRS. The availability of screening programs and well-established ophthalmology clinics in primary healthcare centers in addition to trained physicians will help to overcome the barriers of DRS.

## Introduction

Diabetes mellitus (DM) is estimated to be the seventh cause of death globally. Cardiac diseases, strokes, kidney injuries, limb amputations, and loss of vision are significant complications of diabetes [1]. Saudi Arabia is ranked seventh in the world for the rate of diabetes (24%) [2]. Diabetic patients are counted as around seven million Saudi citizens, while prediabetics are around three million [3].

Diabetic retinopathy (DR) is one of the complications of diabetes that leads to blindness [2,4]. It results in microvascular damage and leads to retinal ischemia and increased vascular permeability [4]. It is divided into A) nonproliferative diabetic retinopathy (NPDR), which is the earlier stage, and B) proliferative diabetic retinopathy (PDR), which is the advanced stage. NPDR is classified based on clinical findings like microaneurysms, retinal hemorrhages, intraretinal microvascular abnormalities, and venous caliber changes. However, pre-retinal neovascularization is the pathological feature of PDR. Diabetic macular edema occurs in both NPDR and PDR and is the most common cause of vision loss in patients diagnosed with DR [5].

The majority of people with diabetes develop some degree of DR and the incidence increases with diabetes duration [2,4]. Other factors that contribute to the development of DR include the type of diabetes, poor glycemic control, hypertension, dyslipidemia, nephropathy, pregnancy, and gender [2,6]. As DR is asymptotic initially, patients do not seek medical help until their vision is impaired and they are in an advanced stage [7]. Frequent diabetic retinopathy screening (DRS) in addition to appropriate and effective treatments can prevent up to 98% of blindness. Moreover, controlling hypertension and dyslipidemia has been reported to improve outcomes [6]. However, lack of knowledge among primary care physicians and patients can lead to poor adherence to regular DRS, affecting patients' quality of life and burdening healthcare systems [2,6]. Other factors like limited access to ophthalmologists, time, finance, and transportation are reported to impact the patients’ adherence in rural areas [8].

As DM and DR emerge as growing problems and pose a high burden on individuals, society, and the healthcare system in Saudi Arabia, this study aimed to assess the level of adherence of diabetic patients to DRS and to identify the influencing factors of adherence among diabetic patients in Al-Ahsa, Saudi Arabia.

## Materials and methods

A cross-sectional study was conducted among diabetic patients from July 11, 2021 to November 7, 2021 in Al-Ahsa, Saudi Arabia. It included diabetic patients with type 1 (T1DM) or type 2 diabetes mellitus (T2DM), aged 18 years and older, attending primary health centers in the Al-Ahsa sectors (Northern, Southern, Eastern, and Central regions). The informed consent was obtained from the patients before filling the questionnaire and the ethical approval was taken from the ethical and research committee of King Fahad Hospital in Hofuf. 

A self-administered questionnaire was adapted from a study by Alwazae M et al. [6]. This questionnaire was divided into five sections: sociodemographic data, diabetic profile, assessment of knowledge, attitude toward DRS, and barriers to DRS. 

Sociodemographic data included age, gender, marital status, level of education, and income, which were assessed by four qualitative answers (enough and save, enough, not enough, and in debt). The second section concerned diabetic profile, comprising type of diabetes, treatment of diabetes, diet, duration of DM, complications of DM, and frequency of screening. Knowledge assessment was based on four questions: Is retinopathy one of diabetes’ complications? Is DR asymptomatic? Are there treatments available for DR? Could regular eye examination prevent the progression of DR? Each question was answered by one of three possible options, either “yes,” “no,” or “I do not know.” Answering more than two questions, the correct answer was considered to have adequate knowledge, while it was inadequate knowledge when answering two questions or less the correct answer. Additional item inquired about the source of knowledge (from physicians, family and friends, and others, including the internet and newspaper). 

The attitude toward DRS was measured on three items related to the importance and benefit of DRS, worrying about blindness due to DR, and attending the screening if requested. A three-point scale was used to assess the response to each item ranging from 1 “agree” to 3 “disagree.” The last section comprised the barriers to DRS like cost, family support, fear of results, lack of knowledge about screening, and screening efficacy. The responses to each barrier were scaled by a three-point scale ranging from 1 “agree” to 3 “disagree.” 

Statistical analysis was done by statistical software IBM SPSS version 22 (IBM Corp, Armonk, NY). All statistical analyses were done using two-tailed tests. A p-value less than 0.05 was statistically significant. For knowledge and awareness items, each correct answer was scored one point and a total summation of the discrete scores of the different items covering general knowledge regarding DR. Participant with a score less than 60% of the total score was considered to have poor awareness level, while good awareness was considered if the score was 60% or more. Descriptive analysis based on the frequency and percentage distribution was done for all variables, including patients’ sociodemographic data, residence sector in Al-Ahsa, diabetes-related data, treatment received, diabetic complications, and eye complications. Also, patients’ awareness and attitude regarding DR and screening were shown in frequency tables alongside reported patient barriers to regular eye screening. Crosstabulation was used to assess the distribution of participants' awareness levels regarding DR according to their personal data, medical data, and source of information. Also, the distribution of patients’ attitudes and reported screening barriers by their residence sector were tabulated. Relations were tested using Pearson's chi-square test and exact probability test for small frequency distributions. 

## Results

A total of 397 diabetic patients fulfilling the inclusion criteria completed the study questionnaire. The majority of the participants were from the Southern region (144; 36.3%) and Northern region (124; 31.2%). Among the participants, 211 (53.1%) patients were females and 282 (71%) were married. As for educational level, 189 (47.6%) patients were university graduates, and 110 (27.75) had below the secondary level of education. The monthly income was just sufficient for 203 patients (51.1%) and more than sufficient for 126 (31.7%). T2DM was reported by 206 (51.95%) of the participants, while 99 (24.9%) had T1DM. Regarding the treatment of DM, 203 (51.1%) were taking oral antidiabetics, 85 (21.4%) were on insulin, and 77 (19.4%) received both of them (Table 1). 

**Table 1:** Bio-demographic data of diabetic patients in Al Ahsa, Saudi Arabia T1DM: type 1 diabetes mellitus; T2DM: type 2 diabetes mellitus; OHG drugs: oral hypoglycemic drugs.

Bio-demographic data	No	%
Residence sector		
Central region	94	23.7
Northern region	124	31.2
Eastern region	35	8.8
Southern region	144	36.3
Gender		
Male	186	46.9
Female	211	53.1
Marital status		
Single	57	14.4
Married	282	71.0
Divorced/widow	58	14.6
Educational level		
Below secondary	110	27.7
Secondary	98	24.7
University/above	189	47.6
Monthly income		
Insufficient	68	17.1
Just sufficient	203	51.1
More than sufficient	126	31.7
Type of diabetes		
T1DM	99	24.9
T2DM	206	51.9
Don't know	92	23.2
Treatment received		
Insulin	85	21.4
OHG drugs	203	51.1
Dietary control	22	5.5
Insulin and OHG drugs	77	19.4
None	10	2.5

Table 2 showed that ocular complications were reported among 140 (35.3%). The most reported eye complications were cataract (37.1%; 52), followed by retinopathy (36.4%; 51). Regarding the frequency of physician visits for eye screening, it was annually among 101 (25.4%) patients, every six months among 77 (19.4%) patients, while 101 (25.4%) patients did not visit physicians for screening.

**Table 2:** General and ocular complications of diabetes among patients in Al Ahsa, Saudi Arabia BGL: blood glucose level; SD: standard deviation.

Complications	No	%
Do you have any diabetes complications?		
Cardiovascular complications	50	12.6
Renal complications	36	9.1
Neurological complications	64	16.1
Oral ulcers/inflammations	47	11.8
None	248	62.5
Do you have any eye disease because of diabetes?		
Yes	140	35.3
No	173	43.6
Don't know	84	21.2
Which of the following eye diseases you have because of diabetes?		
Retinopathy	51	36.4
Cataract	52	37.1
Glaucoma	19	13.6
Macular edema	5	3.6
None	42	30.0
How often do you visit the physician for eye screening?		
Every 3 months	41	10.3
Every 6 months	77	19.4
Annually	101	25.4
Irregularity	77	19.4
None	101	25.4
Last measured BGL		
Range	45-500 mg/dL	
Mean ± SD	158.1 ± 71.6	

Exactly 68% of the patients know that retinopathy is one of the diabetic complications, which was significantly higher among Eastern sector residents (77.1%) than among Northern region residents (64.5%; p = 0.049). Also, 28.2% of the patients know that DR could be an asymptomatic disease, which was highest among Eastern region residents (40%) and lowest among Central region resident patients (21.3%; p = 0.001) (Table 3).

**Table 3:** Diabetic patients’ awareness regarding retinopathy in general and by residence sector in Al Ahsa, Saudi Arabia Statistical difference found by Pearson's chi-square (X2) test is significant as p value is less than 0.05. *p < 0.05 (significant).

Awareness items	Total		Residence sector								p-Value
			Central region		Northern region		Eastern region		Southern region		
	No	%	No	%	No	%	No	%	No	%	
Is retinopathy one of the diabetic complications?											0.049*
Yes	270	68.0%	66	70.2%	80	64.5%	27	77.1%	97	67.4%	
No	18	4.5%	8	8.5%	2	1.6%	2	5.7%	6	4.2%	
Don’t know	109	27.5%	20	21.3%	42	33.9%	6	17.1%	41	28.5%	
Could diabetic retinopathy be asymptomatic disease?											0.001*
Yes	112	28.2%	20	21.3%	41	33.1%	14	40.0%	37	25.7%	
No	84	21.2%	30	31.9%	11	8.9%	8	22.9%	35	24.3%	
Don’t know	201	50.6%	44	46.8%	72	58.1%	13	37.1%	72	50.0%	
Are there available treatments for diabetic retinopathy?											0.574
Yes	170	42.8%	42	44.7%	45	36.3%	17	48.6%	66	45.8%	
No	36	9.1%	6	6.4%	12	9.7%	4	11.4%	14	9.7%	
Don’t know	191	48.1%	46	48.9%	67	54.0%	14	40.0%	64	44.4%	
Could regular eye examination prevent the progression of diabetic retinopathy?											0.106
Yes	270	68.0%	65	69.1%	75	60.5%	29	82.9%	101	70.1%	
No	21	5.3%	7	7.4%	8	6.5%	2	5.7%	4	2.8%	
Don’t know	106	26.7%	22	23.4%	41	33.1%	4	11.4%	39	27.1%	

**Figure 1:**
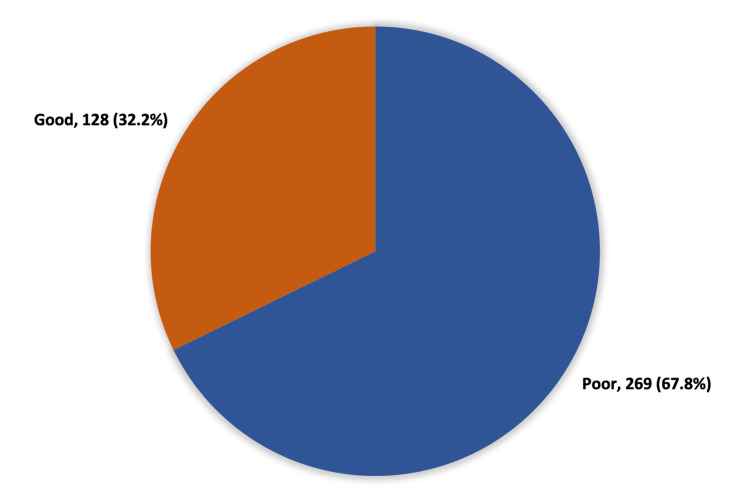
Overall awareness level regarding diabetic retinopathy among diabetic patients in Al-Ahsa, Saudi Arabia

Figure 1 illustrated the overall awareness level regarding DR among diabetic patients in Al-Ahsa, Saudi Arabia. One hundred and twenty-eight (32.2%) patients had a good awareness level regarding DR, while 269 (67.8%) had a poor awareness level. 

Exactly 59.4% of patients worried that they might lose their vision because of diabetes, which was significantly highest among Northern region patients (80.6%) versus 43.6% of Central region patients (p = 0.001). In addition, 90.2% of patients consider regular eye exams to be important, which was significantly highest in patients from the Eastern region (95.7%) and lowest in patients from the Northern region (82.3%; p = 0.002). A total of 96.5% of the patients would undergo an eye screening recommended by the physician, which differs only slightly depending on where they live (Table 4).

**Table 4:** Diabetic patients’ attitude toward retinopathy in general and by residence sector in Al Ahsa, Saudi Arabia Statistical difference found by Pearson's chi-square (X2) test is significant as p value is less than 0.05. ^$^Exact probability test; *p < 0.05 (significant).

Attitude items	Total		Residence sector								p-Value
			Central region		Northern region		Eastern region		Southern region		
	No	%	No	%	No	%	No	%	No	%	
I am worried that I might lose my vision because of diabetes											0.001*
Agree	236	59.4%	41	43.6%	100	80.6%	19	54.3%	76	52.8%	
Neutral	89	22.4%	23	24.5%	21	16.9%	10	28.6%	35	24.3%	
Disagree	72	18.1%	30	31.9%	3	2.4%	6	17.1%	33	22.9%	
I think it is important to have regular eye examination											0.002*^$^
Agree	358	90.2%	90	95.7%	102	82.3%	34	97.1%	132	91.7%	
Neutral	32	8.1%	4	4.3%	20	16.1%	1	2.9%	7	4.9%	
Disagree	7	1.8%	0	0.0%	2	1.6%	0	0.0%	5	3.5%	
If my doctor recommended eye screening for me, I would do it											0.652^$^
Agree	383	96.5%	89	94.7%	120	96.8%	34	97.1%	140	97.2%	
Neutral	10	2.5%	3	3.2%	4	3.2%	1	2.9%	2	1.4%	
Disagree	4	1.0%	2	2.1%	0	0.0%	0	0.0%	2	1.4%	

In Table 5, the most commonly reported barriers were not having any vision or eye problems (50.9%), which was reported by 64.5% of patients from the Northern region versus 36.1% of patients from the Southern region (p = 0.001). The second most reported barrier was difficulty in getting an appointment (50.1%), as reported by 67.7% of patients from the Northern region compared to 36.8% of patients from the Southern region (p = 0.001). The third most common barrier was cost (42.1%), reported by 66.1% of patients in the Northern region versus 25.5% of patients in the Central region (p = 0.001). The fourth barrier was the lack of information about the screening procedure (39.8%), which was among 56.5% of northern region patients compared to 23.6% of Southern region patients (p = 0.001). 

**Table 5:** Reported barriers to diabetic retinopathy screening among diabetic patients in Al Ahsa, Saudi Arabia Statistical difference found by Pearson's chi-square (X2) test is significant as p value is less than 0.05. ^$^Exact probability test; *p < 0.05 (significant).

Barriers	Total		Residence sector								p-Value
			Central region		Northern region		Eastern region		Southern region		
	No	%	No	%	No	%	No	%	No	%	
Cost	167	42.1%	24	25.5%	82	66.1%	11	31.4%	50	34.7%	0.001*
Diseases I have	98	24.7%	16	17.0%	54	43.5%	4	11.4%	24	16.7%	0.001*
Physical disability prevents going for screening	78	19.6%	13	13.8%	52	41.9%	2	5.7%	11	7.6%	0.001*^$^
Lack of time	129	32.5%	30	31.9%	51	41.1%	9	25.7%	39	27.1%	0.133
Lack of family support	92	23.2%	11	11.7%	50	40.3%	9	25.7%	22	15.3%	0.001*
Fear of result	134	33.8%	30	31.9%	57	46.0%	13	37.1%	34	23.6%	0.001*
Having no information about the screening procedure	158	39.8%	43	45.7%	70	56.5%	11	31.4%	34	23.6%	0.001*
Believing the screening is not effective	108	27.2%	17	18.1%	67	54.0%	4	11.4%	20	13.9%	0.001*
Long distance to screening clinic	129	32.5%	27	28.7%	71	57.3%	9	25.7%	22	15.3%	0.001*
Difficult to have an appointment	199	50.1%	45	47.9%	84	67.7%	17	48.6%	53	36.8%	0.001*
I know stable cases with no screening	65	16.4%	22	23.4%	18	14.5%	11	31.4%	14	9.7%	0.006*
I know cases have complications with screening	74	18.6%	22	23.4%	30	24.2%	7	20.0%	15	10.4%	0.054
Lack of physician trust	35	8.8%	6	6.4%	22	17.7%	2	5.7%	5	3.5%	0.001*^$^
Have no visual or eye problem	202	50.9%	49	52.1%	80	64.5%	21	60.0%	52	36.1%	0.001*
Eye examination is painful procedure	84	21.2%	15	16.0%	43	34.7%	6	17.1%	20	13.9%	0.001*
Have controlled BGL	155	39.0%	42	44.7%	50	40.3%	12	34.3%	51	35.4%	0.001*

**Figure 2:**
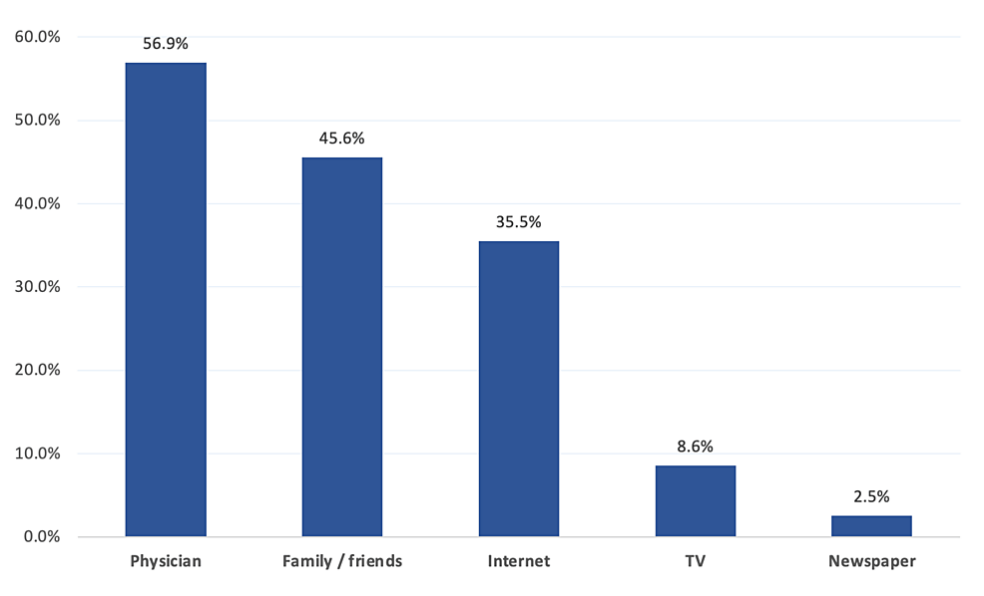
Source of knowledge regarding diabetic retinopathy among diabetic patients in Al-Ahsa, Saudi Arabia

The most reported source of patients’ awareness regarding DR was a physician (56.9%), followed by family/friends (45.6%) and the internet (35.5%) (Figure 2).

Exactly 41.4% of male patients had a good awareness level compared to 24.2% of females with statistical significance (p = 0.001). Also, 36.9% of married patients had a good awareness level versus 19% of the divorced group (p = 0.008). A total of 38.6% of patients with diabetes-related eye diseases had good awareness regarding retinopathy in comparison to 19% of those with no idea (p = 0.009). Good awareness was detected among 48.1% of patients who visit doctors every six months versus 19.5% with irregular visits (p = 0.001) (Table 6).

**Table 6:** Distribution of diabetic patients’ awareness regarding retinopathy by their bio-demographic data and source of information T1DM: type 1 diabetes mellitus; T2DM: type 2 diabetes mellitus. Statistical difference found by Pearson's chi-square (X2) test is significant as p value is less than 0.05. *p < 0.05 (significant).

Factors		Awareness level				p-Value
		Poor		Good		
		No	%	No	%	
Residence sector	Central region	67	71.3%	27	28.7%	0.344
	Northern region	88	71.0%	36	29.0%	
	Eastern region	20	57.1%	15	42.9%	
	Southern region	94	65.3%	50	34.7%	
Gender	Male	109	58.6%	77	41.4%	0.001*
	Female	160	75.8%	51	24.2%	
Marital status	Single	44	77.2%	13	22.8%	0.008*
	Married	178	63.1%	104	36.9%	
	Divorced/widow	47	81.0%	11	19.0%	
Educational level	Below secondary	80	72.7%	30	27.3%	0.209
	Secondary	69	70.4%	29	29.6%	
	University/above	120	63.5%	69	36.5%	
Monthly income	Insufficient	54	79.4%	14	20.6%	0.077
	Just sufficient	132	65.0%	71	35.0%	
	More than sufficient	83	65.9%	43	34.1%	
Type of diabetes	T1DM	62	62.6%	37	37.4%	0.119
	T2DM	137	66.5%	69	33.5%	
	Don't know	70	76.1%	22	23.9%	
Do you have any eye disease because of diabetes?	Yes	86	61.4%	54	38.6%	0.009*
	No	115	66.5%	58	33.5%	
	Don't know	68	81.0%	16	19.0%	
How often you visit the physician for eye screening?	Every 3 months	23	56.1%	18	43.9%	0.001*
	Every 6 months	40	51.9%	37	48.1%	
	Annually	67	66.3%	34	33.7%	
	irregularly	62	80.5%	15	19.5%	
	None	77	76.2%	24	23.8%	
Source of information regarding retinopathy	Physician	141	62.4%	85	37.6%	0.056
	Family/friends	129	71.3%	52	28.7%	
	Internet	92	65.2%	49	34.8%	
	TV	20	58.8%	14	41.2%	
	Newspaper	9	90.0%	1	10.0%	

## Discussion

DR is a common complication of both T1DM and T2DM resulting from microvasculature damage in the retina with an estimated prevalence equal to 34.6% globally. DR is considered as a leading cause of vision loss among patients aged 20-74 years and can be prevented up to 90% with early treatment. Adherence to DR screening is important to diagnose and prevent DR [9,10]. This study aimed to assess the diabetic patients’ adherence to DR screening and to identify the barriers affecting their adherence to it in Al-Ahsa, Saudi Arabia. 

In this paper, ocular complications were reported among 35.3% of the respondents, including cataract, retinopathy, glaucoma, and macular edema (37.1%, 36.4%, 13.6%, and 3.6%; respectively). Lower percentages were recorded by Alwazae M, et al. including cataracts (31.2%), DR (20%), glaucoma (6.2%), and macular edema (2.5%) [6]. However, a study conducted by Al-Esawi et al. reported a higher result regarding retinopathy (63.21%) among type 2 diabetic male patients. [11].

The adherence to eye screening was inadequate, as only 219 (55.1%) of the participants had annual or frequent visits to the physician for eye screening compared to those who irregularly had it or did not do it previously (178, 44.8%). Alzahrani SH et al. found that 35% of their participants did not go to their eye checkups [2]. On the other hand, Alwazae M et al. reported that 61.4% of their subjects were attending the screening [6].

The majority of the respondents in this paper showed poor awareness levels regarding DR (67.8%) in contrast to those who had good awareness levels (32.2%). While 77.1% of Eastern sector residents were aware that retinopathy is one of the diabetic complications, the lowest percentage was recorded among the Northern region residents (64.5%) (p = 0.049). The knowledge that DR could be asymptomatic was the highest among Eastern region residents (40%) in comparison to the lowest among Central region resident patients (21.3%) (p = 0.001). However, no significant relation was found between the level of awareness and residence sectors (p = 0.344).

Alwazae M, et al. reported that half of their respondents (49%) had adequate knowledge about DR while a higher percentage was reported in another study done in Oman (72%) [6]. A lower result was found by Venugopal D et al., in which 34.9% of the subjects were aware of DR and 34.1% of them had adequate knowledge. They revealed that awareness and knowledge of DR were significantly related to the level of education. In addition, no significant association was found between knowledge of DR, gender, and socioeconomic status [12]. In our study, both education and monthly income were not significantly related to the knowledge about DR while a statistical significance was found between awareness and gender, marital status, visiting the physician, and having eye disease due to diabetes (p < 0.05). 

Good attitude had been shown by our participants; 59.4% of them worried about losing vision because of diabetes. While 90.2% of them considered the significance of having regular eye screening, 96.5% of the participants would undergo eye screening if it was recommended by the physician. The differences in attitude between the sectors were significant (p < 0.05) regarding worrying about loss of vision and the importance of doing eye screening and were insignificant in responding to the physician's recommendation to do the eye screening (p = 0.652). Similar findings were reported by Alwazae M et al., as 66.8% were worried about blindness due to DR, 96% agreed to do the screening if it was requested by a physician, and 97% considered the importance of screening [6].

The most identified barriers that were reported by all the participants were having no visual or eye problems, difficulty having an appointment, the cost, and lack of information about the screening procedure (50.9%, 50.1%, 42.1%, 39.8%, respectively). These barriers were more reported in the Northern sector (64.5%, 67.7%, 66.1%, and 56.5%, respectively). In the Southern sector, difficulty in having an appointment, having no visual or eye problems, having controlled blood sugar level, and the cost were the most commonly reported barriers (36.8%, 36.1%, 35.4%, 34.7%, respectively). Having no visual or eye problems and difficulty in having appointments were also recorded as the highest percentages among the participants in both the Eastern region (60.0% and 48.6%) and in the Central region (52.1% and 47.9%) in addition to fear of the results (37.1%) in Eastern region. 

Alwazae M et al. revealed that financial barriers, lack of knowledge, asymptomatic nature of DR, low educational attainment and poor literacy, lack of awareness, and time and priority issues were recorded as the major barriers among their participants (40%, 25%, 21%, 21%, 20%, 16%, respectively) [6]. Kumar S et al. reported that patients did not seek eye care unless there was eye discomfort or pain. In addition, fear of having more medicines, costs, managing the dosage, traveling to seek eye care, inappropriate communication between the doctor and the patient, and lack of time and motivation were considerable factors reported by their participants [13]. Lu Y et al. investigated the barriers to DR screening among patients and care providers in Los Angeles, California, and found that financial burdens and depression were the most reported by the participants (26% and 22%, respectively) while both were reported by 14%. In addition, lack of transportation, lack of time, and language issues were reported by 15% of their participants [14].

In a rural community, patients need to travel for a prolonged time and long distances, with difficulties in transportation. They complained about the limited access to health care and limited availability of healthcare providers. In addition, they prioritized acute medical issues over preventive care due to limited resources, time, energy, and money as they have multiple health conditions. Family members suffering from DM complications caused participants to fear the screening results, leading to avoiding diabetic eye screening. However, some of them were motivated to have an annual diabetic eye screening to prevent complications [8].

Our findings can be attributed to and influenced by the number of respondents from each sector, as there was a remarkable variation. The type of sector is also an important factor, whether the sector is rural or urban. However, some of them (including the Northern and Southern sectors) have both rural and urban areas.

In a study by Sahu S et al. to assess the knowledge and awareness of ocular diseases and eye health among the rural and urban communities of the Siraha district, Nepal, a high level of knowledge and awareness was found in the urban community compared to the rural community. However, their subjects showed a low level of awareness and knowledge regarding glaucoma and DR in both urban and rural communities [15]. Another study conducted by Assem AS et al. among diabetic patients showed that being an urban resident was positively associated with good knowledge of DR, which contributed to good practice including regular eye checkups [16]. 

## Conclusions

The findings of this paper showed inadequate adherence to eye screening and poor level of awareness among study participants regardless of residential sectors. On the other hand, a good attitude had been shown by the majority. Having no visual or eye problems, difficulty to have an appointment, the cost, and lack of information about the screening procedure were the most reported barriers among the participants. Improving the patients’ knowledge and awareness is an important step in enhancing their adherence to DRS and reducing the risk of DR and its complications via health campaigns and sufficient education during clinical visits. The barriers also can be overcome by establishing screening programs and affordable ophthalmology clinics in primary healthcare centers, in addition to good training for general practitioners and family physicians to examine diabetic patients.
